# EIF4A3-induced circFAT1 promotes high glucose-induced podocyte damage via miR-30e-5p/SOX4 axis

**DOI:** 10.1080/15476286.2025.2563865

**Published:** 2025-10-20

**Authors:** Youqun Huang, Yu Liu, Mengfan Yang, Liangbin Zhao, Ming Chen

**Affiliations:** a Department of Nephrology, Hospital of Chengdu University of Traditional Chinese Medicine, Chengdu, Sichuan, China; b Department of Nephrology, Deyang Hospital Affiliated Hospital of Chengdu University of Traditional Chinese Medicine, Deyang, Sichuan, China

**Keywords:** Diabetic nephropathy, circFAT1, epithelial-mesenchymal transition, migration, SOX4

## Abstract

Podocyte injury significantly contributes to glomerular filtration dysfunction and albuminuria in diabetic nephropathy (DN). Circular RNAs, particularly circFAT1 (hsa_circ_0001461), have emerged as influential regulators in pathological processes. This research focused on exploring the function of hsa_circ_0001461 in high glucose (HG)-induced podocyte damage and the associated underlying mechanism. Here, we demonstrate that circFAT1 is significantly upregulated in HPCs under HG conditions. Inhibition of circFAT1 led to decreased podocyte migration and a restoration of differentiation markers, along with a reduction in mesenchymal markers. Mechanistically, circFAT1 was found to inhibit miR-30e-5p, resulting in enhanced SOX4 expression, which promoted epithelial-mesenchymal transition and migration in podocytes. Moreover, we identified EIF4A3 as a crucial regulator of circFAT1 biogenesis under hyperglycaemic conditions. Importantly, elevated levels of circFAT1 were also detected in DN patients, correlating with increased albuminuria and serum creatinine. In conclusion, this study elucidates the critical role of circFAT1 in HG-induced podocyte injury through the miR-30e-5p/SOX4 signalling pathway. The findings suggest that targeting circFAT1 May offer a potential strategy for DN intervention.

## Introduction

Diabetic nephropathy (DN), a serious complication of diabetes mellitus, is a leading cause of end-stage renal disease. Podocyte damage is one of the important characteristics during the pathogenesis and progression of DN. Podocytes, which are highly specialized glomerular epithelial cells situated outside the glomerular basement membrane, are involved in the formation of the glomerular filtration barrier alongside the basement membrane and endothelial cells [1]. In the progression of DN, podocyte damage or detachment from the glomerular basement membrane frequently results in a reduced number of podocytes. This disruption compromises the structural integrity of the glomerular filtration barrier, ultimately resulting in albuminuria [2,3]. Therefore, exploring the mechanism of podocyte injury is vital for an deep understanding of the pathological mechanism of DN.

Epithelial-mesenchymal transition (EMT) represents the early phase of podocyte injury, leading to increased podocyte motility, proteinuria, and glomerular fibrosis [4]. In the process of EMT, podocytes lose the specific markers of mature podocytes, including Wilms’ Tumor 1 (WT-1), Nephrin, ZO-1, and P-cadherin. In contrast, mesenchymal cell markers such as fibroblast-specific protein-1 (FSP-1), Desmin, and α-SMA were increased [4]. Phenotypic alteration of podocytes results in podocyte dysfunction and filtration barrier damage, ultimately facilitating the progression of DN.

Circular RNAs (circRNAs) are endogenous non-coding RNAs with circular structure [5]. Evidence confirmed that circRNAs are involved d in the pathogenesis of kidney diseases, including DN [6–8]. For example, Liu F et al. [9] revealed that circHIPK3 aggravates podocyte injury and diabetic kidney disease progression by the FUS/EDA2R axis. Circ-FBXW12 promoted DN progression via modulating miR-31-5p/LIN28B axis [10]. CircFAT1 (hsa_circ_0001461) is back-spliced from exon 2 of the FAT1 pre-mRNA. Accumulating evidence indicates that circFAT1 is an important regulator in various human diseases, including cancers and diabetic retinopathy [11–13]. However, the potential role and mechanism of circFAT1 in DN remains obscure.

Herein, we profiled the expression of circFAT1 in DN patients and HG-treated podocyte. Functional experiments demonstrated the promotive role of circFAT1 in HG-induced podocyte apoptosis, migration, and EMT. In addition, we also uncovered the molecular mechanism of circFAT1.

## Methods

### Cells and cell culture

Human podocyte cells (HPCs, #BNCC340460; Bena, Suzhou, China) were cultured in RPMI 1640 medium. Following a 12-hour serum starvation period, cells were exposed to three different conditions for 48 h: normal glucose (NG, 5.6 mM glucose), hyperosmotic medium (HM, 5.6 mM glucose +24.4 mM mannitol), and high glucose (HG, 30 mM glucose).

### Transfection

CircFAT1 small interfering RNA (si-circFAT1) and the control (si-NC), miR-30e-5p mimics and the control (mimic NC), miR-30e-5p inhibitor and the control (inhibitor NC), SOX4 overexpression vector (OE_SOX4) and the empty vector (Vector) were obtained from Hanbio (Shanghai, China). Transfection was conducted with Lipofectamine 2000 (#11668019, ThermoFisher, Shanghai, China).

### Cell viability assay

HPCs (1 × 10^3^ cells/100 μL) were seeded into 96-well plates. After 48 h treatments, 10 μL CCK-8 reagent was added to each well. Plates were incubated at 37°C for 2 hours in the dark. Absorbance was measured at 450 nm using a microplate reader.

### Transwell migration assay

HPCs were inoculated into the upper chamber of Transwell, and 600 μl of culture medium (containing 10% foetal bovine serum) was added to the lower chamber. After 24 hours of culture, the cells were fixed with 70% ethanol, stained with 1% crystal violet, and the number of invasive cells was counted and photographed for record.

### Dual luciferase assay

The binding sites of miR-30e-5p with circFAT1 and SOX4 were mutated. The luciferase reporter gene vectors were respectively loaded at the binding sites and the mutation sites to construct wild-type vectors (WT-circFAT1, WT-SOX4) and mutant vectors (MUT-circFAT1, MUT-SOX4). Then, WT-circFAT1, WT-DNMT3A, MUT-circFAT1, and MUT-SOX4 were co-transfected with miR-138-5p mimics and miR-NC into cancer cells, and the relative luciferase activity was detected after 48 hours of culture.

### Quantitative real-time PCR (qRT-PCR)

Total RNAs was extracted from HPCs using TRIzol (#R0016, Beytime, Shanghai, China). 1 μg of total RNAs were reverse transcribed to cDNA as a PCR template. QRT-PCR was carried out utilizing the IQ SYBR Green SuperMix (#1708880, Bio-Rad, Hercules, CA, USA). U6 was used employed as the internal control of miR-30e-5p, while GAPDH for circFAT1 and SOX4. The following primer sequences were used: circFAT1, forward: GCTCTGGCGTTGGTGTTTTC and reverse: GCTGCCATCACTGTCTCCAA; FAT1, forward: CCTTCCAACAGCCACATCCACTAC and reverse: TTGAACCGTGAGCGTGTAACCTG; SOX4, forward: CCGAGCTGGTGCAAGACC and reverse: CCACACCATGAAGGCGTTC; GAPDH, forward: TATGATGACATCAAGAAGGTGGT and reverse: TGTAGCCAAATTCGTTGTCATAC; GAPDH, forward: TATGATGACATCAAGAAGGTGGT and reverse: TGTAGCCAAATTCGTTGTCATAC; miR-30e-5p, forward: GCGCGTGTAAACATCCTTGAC and reverse: AGTGCAGGGTCCGAGGTATT; U6, forward: CTCGCTTCGGCAGCACA and reverse: AACGCTTCACGAATTTGCGT.

### Western blot

Cells were lysed using protein extraction buffer, a subjected to SDS-PAGE and then transferred to nitrocellulose membranes. Membranes were blocked with 5% non-fat milk in TBST, followed by incubation with specific primary antibodies. The following antibodies were utilized: WT-1 antibody (#12609–1-AP, 1: 1000), Nephrin antibody (#66970–1-Ig, 1:1000), FSP1 antibody (#20886–1-AP, 1:5000) and Desmin antibody (#67793–1-Ig, 1:5000) and GAPDH antibody (#60004–1-Ig 1:10000) were obtained from Proteintech (Wuhan, China). After washing with TBST, membranes were incubated with appropriate secondary antibodies, then washed again before detection.

### Flow cytometry

Cell apoptosis was assessed using Annexin V-FITC/PI Apoptosis Detection Kit (#40302E50, Yeasen, Shanghai, China) following the manufacturer’s protocols. Samples were loaded on a BD FACSCalibur (Becton Dickinson, NJ, USA) and analysed by Flow Jo.

### Immunofluorescence

Podocytes were plated on glass slides in a 24-well plate and cultured for 48 hours. Subsequently, the cells were fixed and permeabilized. Following this, the cells were incubated with 1% bovine serum albumin. (# A600332, Sangon Biotech, Shanghai, China) and then treated with the following specific primary antibodies: WT-1 polyclonal antibody (#12609–1-AP, 1: 1000), Nephrin monoclonal antibody (#66970–1-Ig, 1:1000), FSP1 polyclonal antibody (#20886–1-AP, 1:5000) and Desmin monoclonal antibody (#67793–1-Ig, 1:5000) overnight at 4°C. Following PBS rinses, the sections were subsequently incubated with the secondary antibody. After that, the cells were stained with DAPI (#A606584, Sangon Biotech, Shanghai, China) and examined using an inverted fluorescence microscope.

### Dual-luciferase assay

Dual-luciferase assay was conducted to confirm the relationship between miR-30e-5p and circFAT1 or SOX4. The wild-type and mutant luciferase reporter plasmids for circFAT1 and SOX4 were constructed by Jikai (Shanghai, China). These luciferase plasmids were co-transfected into 293T cells using Lipofectamine 2000 with miR-30e-5p mimics or control. Following a 48-hour incubation, luciferase activity were detected using the Dual-Luciferase Reporter Assay System (catalogue #N1610, Promega, Wisconsin, USA).

### Clinical specimens

Thirty-eight patients with pathological diagnosis of DN were recruited from May 2018 to June 2021. The detailed clinical information for these DN patients is presented in Table 1. Thirty-eight healthy controls were recruited from the same hospital during the same period. The mean age was 50.3 years, with 18 males and 20 females. Urine samples from 38 DN patients and 38 healthy controls were collected for follow-up detection and analysis in the single-blind condition. This study was approved by the Ethics Committee of the Hospital of Chengdu University of Traditional Chinese Medicine (Approval No. 2022KL–046). Written informed consent was obtained from all participants, and the study was conducted in accordance with the principles outlined in the Declaration of Helsinki.

**Table 1.** Characteristics of DN patients for urine collection and RT-PCR analysis.

Variables	Low circFAT1 group (*n* = 19)	High circFAT1 group (*n* = 19)	*P* value
Age(yrs), Mean ± SD	48.0 ± 3.1	48.8 ± 4.6	1.000
Gender (Male/Female)	9/10	11/8	0.892
Glucose (mmol/L), Mean ± SD	9.9 ± 3.7	9.3 ± 2.2	0.604
SCr (µmol/L) Mean ± SD	128.6 ± 15.3	140.0 ± 16.7	**0.035**
SUN (µmol/L) Mean ± SD	8.0 ± 2.9	7.9 ± 1.8	0.894
CysC (mg/L) Mean ± SD	1.2 ± 0.3	1.5 ± 0.4	**0.014**
eGFR (mL/min·1.73 m^2^ mg/L) Mean ± SD	45.9 ± 6.5	40.8 ± 7.8	0.033
24-h urine protein (g/d) Mean ± SD	1.4 ± 0.3	1.8 ± 0.4	0.180

SCr, serum creatinine; SUN, serum urea nitrogen; CysC, cystatin-C; eGFR, estimated glomerular filtration rate.

### Statistical analysis

Data were shown as means ± standard deviation. Statistical analysis used Student’s t-test, one-way ANOVA, or two-way ANOVA. *p* < 0.05 was considered statistically significant. Multivariate linear regression models were constructed using SPSS 26.0, with circFAT1 level as the independent variable and DN severity markers (albuminuria, SCr, eGFR) as dependent variables. Covariates included age, gender, hypertension (defined as BP > 140/90 mmHg or antihypertensive use), and dyslipidemia (LDL > 130 mg/dL or lipid-lowering therapy)

## Results

### Elevated expression of circFAT1 in HG-treated HPCs

**Figure 1.**
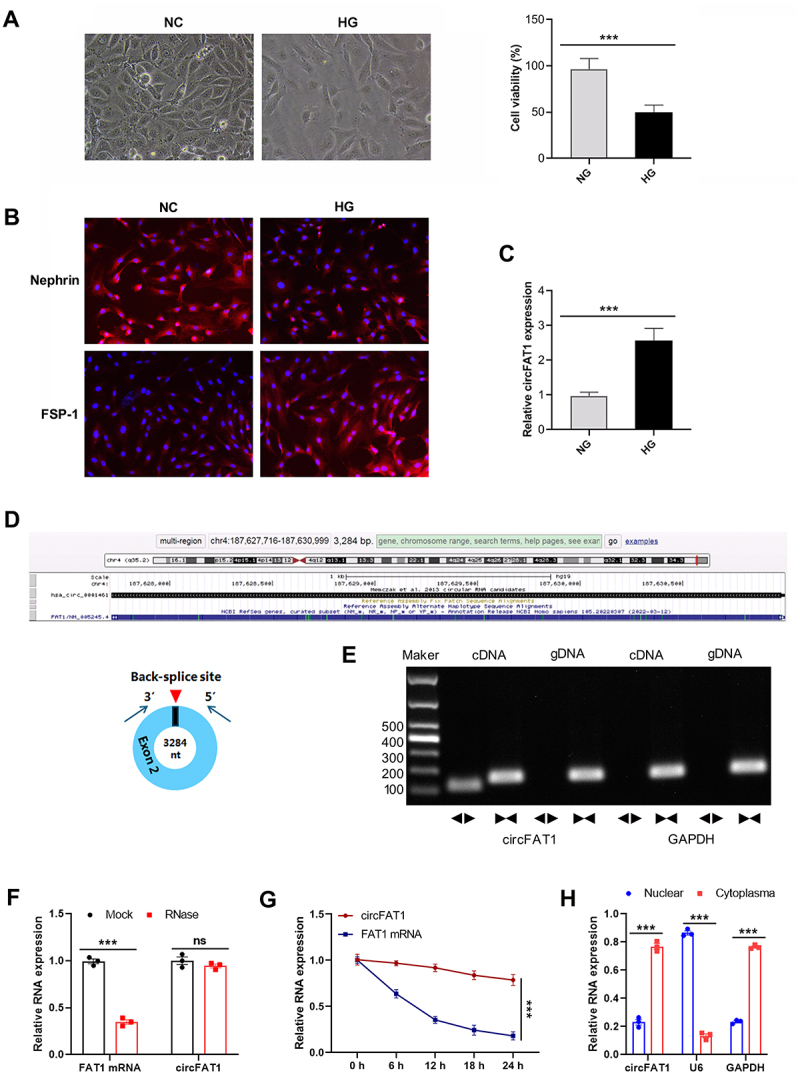
Elevated expression of circFAT1 in HG-treated HPCs. (A) cell morphology (left) and viability measured by CCK-8 assay (right) after 48 h treatments. (B) immunofluorescence staining of Nephrin and FSP1. (C) CircFAT1 expression by qRT-PCR. (D) schematic diagram of the composition of circFAT1. (E) PCR-agarose gel electrophoresis was used to verify the back splicing sequence and RT-PCR product of circFAT1. (F) the circFAT1 and FAT1 mRNA abundance after RNase R treatment was determined via qRT-PCR. (G) RNA stability after actinomycin D (5 μg/mL) treatment. (H) nuclear and cytoplasmic fractionation showed the distribution of circFAT1 in HPCs. ***p < 0.001.

HPCs were treated with HG, and CCK-8 assay revealed that HG treatment resulted in reduced cell viability of HPCs (Figure 1(A)). The immunofluorescence staining demonstrated decreased Nephrin, a key slit diaphragm protein, and increased FSP1, a marker of podocyte injury and epithelial-mesenchymal transition (EMT), in HG-treated HPCs (Figure 1(B)). Then, we determined circFAT1 expression in HPCs under NG or HG condition. The qRT-PCR analysis showed significantly elevated circFAT1 levels in HG-treated HPCs compared to normal glucose (NG) conditions (Figure 1(C)), implicating that circFAT1 May be involved with HG-Treated HPC dysfunction.

CircFAT1, from the exon 2 of the FAT1 pre-mRNA, comprises 3,283 nucleotides (Figure 1(D)). First, specific divergent and convergent primers were designed for circFAT1 and circFAT1 pre-RNA, respectively. PCR amplification was performed using gDNA and cDNA as templates. The results showed that circFAT1 was detectable only in cDNA, not in gDNA, while circFAT1 pre-RNA could be identified in both cDNA and gDNA (Figure 1(E)), indicating its circular nature. Furthermore, RNase R digestion reduced linear FAT1 mRNA but not circFAT1 levels (Figure 1(F)), underscoring its stability. Treatment with the transcriptional inhibitor actinomycin D (5 μg/mL) revealed a significantly longer half-life for circFAT1 compared to linear FAT1 mRNA (Figure 1(G)). Besides, nuclear/cytoplasmic fractionation experiment revealed that circFAT1 predominantly localized in the cytoplasm (Figure 1(H)).

### Depletion of circFAT1 attenuates HG-induced migration and EMT in podocytes

**Figure 2.**
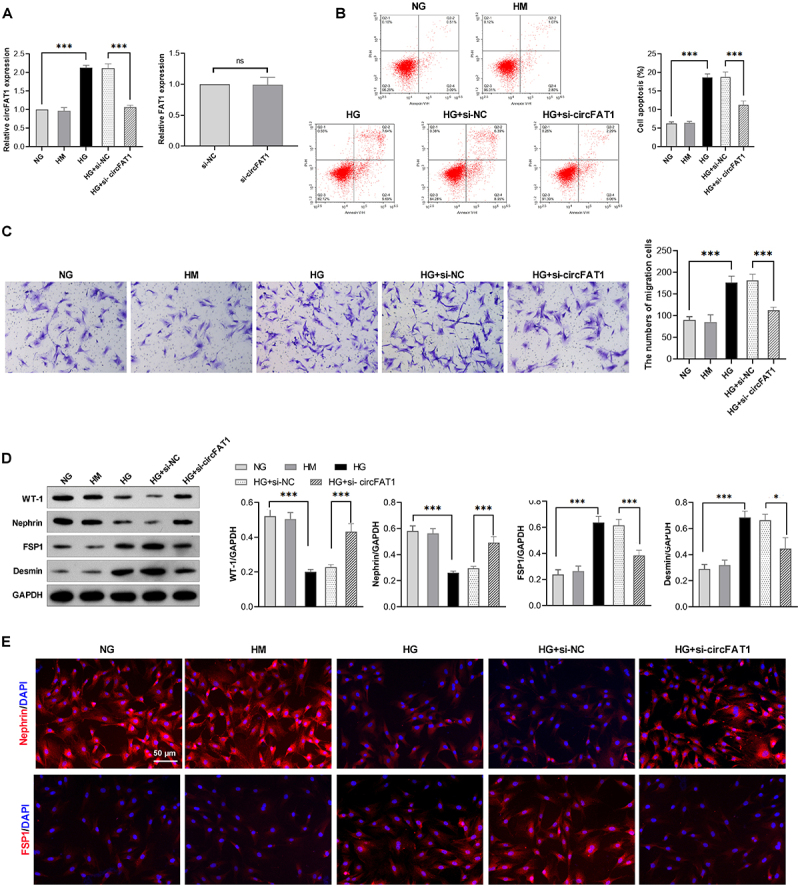
Depletion of circFAT1 attenuates HG-induced podocyte migration and EMT. (A) levels of circFAT1 and FAT1 in HPCs transfected with si-circFAT1. (B) the apoptosis of HPCs was determined using flow cytometry. (C) the motility of HPCs was determined using Transwell assays. (D) Western blotting demonstrated the expression of WT-1, Nephrin, FSP1, and Desmin under different conditions. (E) immunofluorescence staining of Nephrin and FSP1. Bar = 50 μm, *p < 0.05, ***p < 0.001.

To assess the role of circFAT1 in HPC injury, siRNAs were used to downregulate circFAT1 in HG-treated HPCs, and qRT-PCR confirmed successful knockdown. Moreover, linear FAT1 mRNA levels remained unchanged (Figure 2(A)). Flow cytometry results showed that HG treatment induced apoptosis in HPCs, which was alleviated by the knockdown of circFAT1 (Figure 2(B)). Moreover, Transwell assays indicated that HG treatment enabled HPC migration, whereas circFAT1 knockdown significantly suppressed the promotive effect of HG on the migration of HPCs (Figure 2(C)).

EMT is the main pathway to HPC dysfunction, proteinuria, and glomerulosclerosis [14]. Therefore, the mature podocyte markers WT-1 and Nephrin expression and the mesenchymal cell markers FSP1 and Desmin were measured. As Figure 2(D), western blot analysis showed that HG treatment reduced WT-1 and Nephrin expression, while promoting the expression of FSP1 and Desmin. However, gene silencing of circFAT1 could recover the expressions of WT-1 and Nephrin and reduce the expression of FSP1 and Desmin in HG-treated podocytes. Moreover, the immunofluorescence staining further revealed similar changes of Nephrin and FSP1 in podocytes (Figure 2(E)). These results indicate that depletion of circFAT1 could mitigate the dedifferentiation of HG-induced podocyte.

### CircFAT1 enhanced SOX4 expression by directly interacting with miR-30e-5p

**Figure 3.**
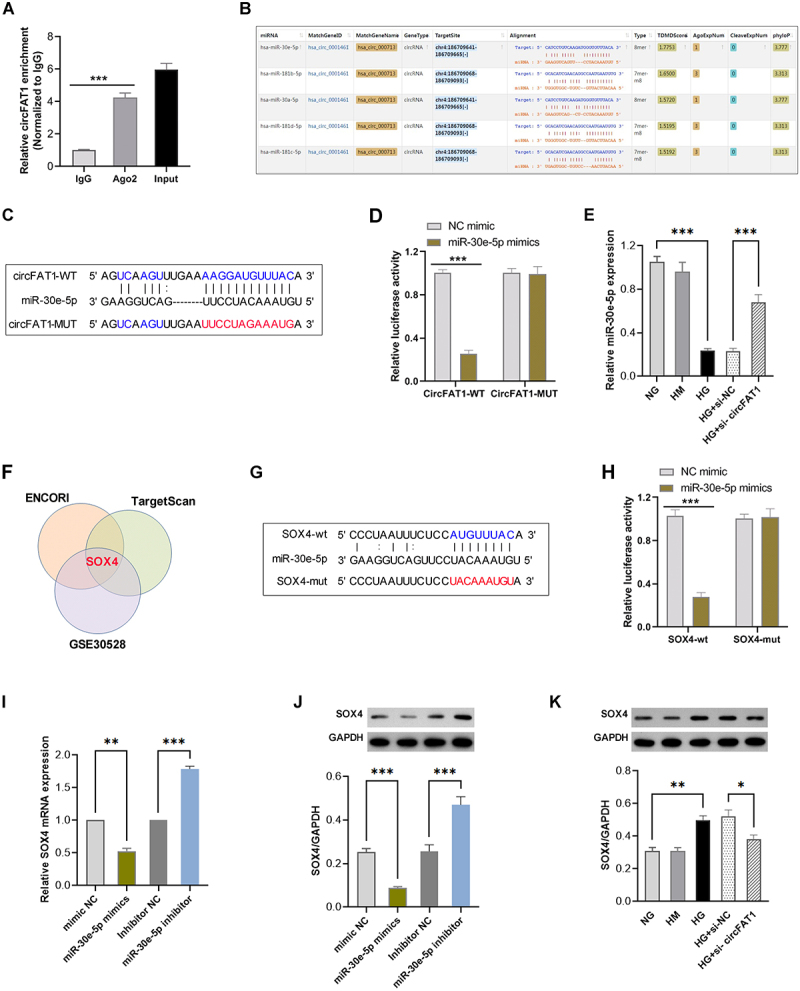
circFAT1 mediated SOX4 expression by associating with miR-30e-5p. (A) RIP assay was conducted to analyse the abundance of circFAT1 by Ago2 antibody. (B) miR-30e-5p was predicted by the ENCORI database to contain possible binding sites for circFAT1. (C) the wild type (circFAT1-WT) and mutant (circFAT1-MUT) luciferase reporter plasmids for circFAT1 were constructed. (D) dual-luciferase reporter assay was conducted to analyse the interaction between miR-30e-5p and circFAT1. (E) the expression of miR-30e-5p in HPCs co-treated with circFAT1 knockdown and HG or alone. (F) venn diagram of miR-30e-5p targets from ENCORI, TargetScan, and GSE30528. (G) the complementary binding site of SOX4 for miR-30e-5p. (H) luciferase activity of SOX4 reporters with miR-30e-5p mimics. (I) SOX4 mRNA levels by qRT-PCR. (J) SOX4 protein levels by Western blot. (K) SOX4 protein after circFAT1 knockdown. *p < 0.05, **p < 0.01, ***p < 0.001.

The Circular RNA Interactome website showed that AGO2, which is the crucial protein of the RNA-induced silencing complex (RISC) [15], was the potential RNA-binding protein of circFAT1. RIP assay confirmed that circFAT1 were significantly enriched by Ago2 immunoprecipitation complex (Figure 3(A)), indicating that circFAT1 May competitively bind miRNAs in a ceRNA-like manner. Through the ENCORI online database, we found that miR-30e-5p, which has been reported to be downregulated in DN patients, was predicted to be likely to bind to circFAT1 (Figure 3(B)). Then, according to the predicted binding sites of circFAT1 binding to miR-30e-5p, the wild type (circFAT1-WT) and mutant (circFAT1-MUT) luciferase reporter plasmids for circFAT1 were constructed (Figure 3(C)). The luciferase assay demonstrated that miR-30e-5p mimics significantly reduced the luciferase activity of circFAT1-WT plasmids. In contrast, no significant change was seen in the luciferase activity of circFAT1-MUT plasmids (Figure 3(D)). Besides, the expression of miR-30e-5p was lower in HG-induced HPCs than that in the NG group, while circFAT1 knockdown partially restored miR-30e-5p expression (Figure 3(E)). These data suggest that circFAT1 binds to miR-30e-5p and inhibits its expression of in HPCs.

Next, ENCORI and HumanTargetScan were utilized to analyse the potential targets of miR-30e-5p, and 1223 mRNAs were screened. By overlapping these genes with these up-regulated differential expression genes in the DN from the GSE30528 dataset, SOX4 was filtered out to be the target of miR-30e-5p (Figure 3(F)). We constructed the luciferase reporter plasmids based on the complementary binding site of SOX4 for miR-30e-5p (Figure 3(G)). Next, luciferase assay confirmed that miR-30e-5p mimics reduced the luciferase activity of the SOX4-wt group, whereas the relative luciferase activity in the SOX4-mut group remained unaffected (Figure 3(H)). Additionally, the expression level of SOX4 was inversely correlated with miR-30e-5p expression (Figure 3(I–J)). In addition, HG elevated the SOX4 protein level, which was significantly inhibited following the transfection of si-circFAT1 (Figure 3(K)). Collectively, these data displayed that circFAT1 promotes SOX4 expression by sponging miR-30e-5p.

### Overexpressing SOX4 reversed the protective effect of silencing circFAT1 on podocytes

**Figure 4.**
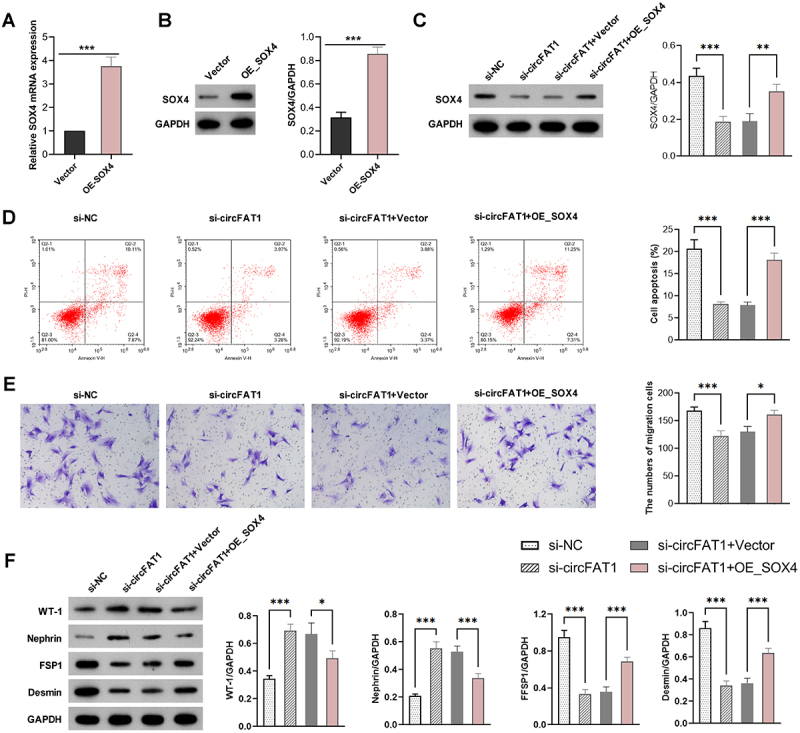
SOX4 overexpression attenuated the protective effect of circFAT1 knockdown against HG-induced podocyte damage. (A) SOX4 mRNA expression in HPCs transfected with SOX4-expressing plasmids. ***p < 0.001. (B) SOX4 protein expression in HPCs transfected with SOX4-expressing plasmids. ***p < 0.001 (C) SOX4 protein expression in HPCs transfected with NC, si-circFAT1, si-circFAT1 + Vector or si-circFAT1 + SOX4_oe, under HG condition. (D) apoptosis by annexin V/PI staining. (E) migration by Transwell assay. (F) protein levels of podocyte (WT-1, Nephrin) and mesenchymal (FSP1, Desmin) markers. *p < 0.05, **p < 0.01, ***p < 0.001.

SOX4-expressing plasmids were transfected into HPCs, and PCR and Western blotting confirmed the upregulation of SOX4 (Figure 4(A–B)). To assess the role of SOX4 in circFAT1-mediated biological function, SOX4-expressing plasmids and si-circFAT1 were co-transfected into HPCs under HG conditions. Western blotting showed that si-circFAT1 reduced SOX4 expression, which was reversed by SOX4-expressing plasmids (Figure 4(C)). Moreover, FCM and transwell assays revealed that knockdown of circFAT1 dramatically blocked HG-induced apoptosis and migration, while these protective effects were abrogated by the overexpression of SOX4 (Figure 4(D–E)). In addition, the knockdown of circFAT1 up-regulated the expression of WT-1 and Nephrin while restraining FSP1 and Desmin expression (Figure 4(F)) in HPCs under HG, whereas these effects were eliminated by SOX4 overexpression. In summary, these outcomes indicate that circFAT1 affects the apoptosis, migration, and EMT of HPCs under HG by regulating SOX4.

### EIF4A3 promotes the expression of circFAT1

**Figure 5.**
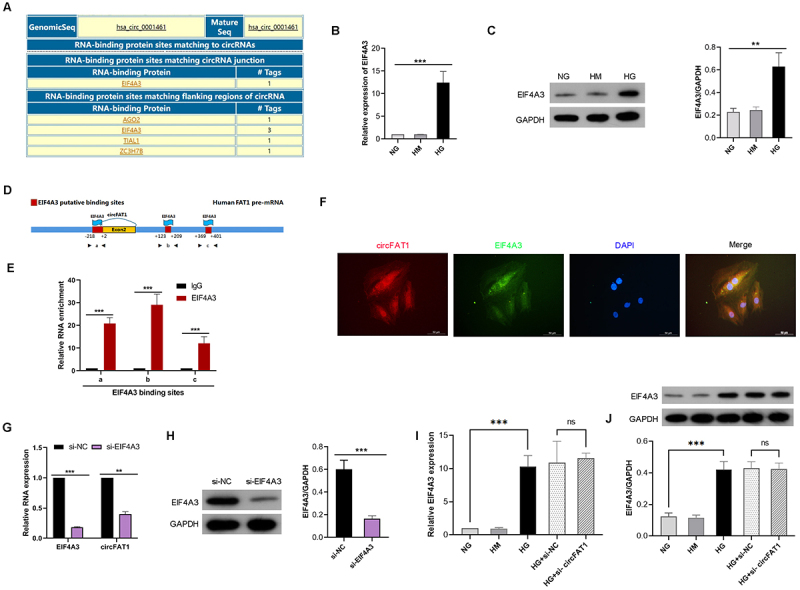
EIF4A3 promotes the expression of circFAT1. (A) predicted RNA-binding proteins for circFAT1 (CircInteractome). (B) QRT-PCR and (C) Western blot were used to detect the mRNA and protein expression of EIF4A3 in HPCs after normal glucose (NG), hyperosmotic solution (HM), or high glucose (HG) treatment for 48 h. (D) schematic of EIF4A3 binding sites in circFAT1 pre-mRNA. (E) RIP-qPCR enrichment of circFAT1 regions with anti-EIF4A3 antibody. (F) FISH and Immunofluorescence staining were conducted to observe the colocalization of circFAT1 and EIF4A3. (G) QRT-PCR analysis of EIF4A3 and circFAT1 in HPCs transfected with si-EIF4A3. (H) Western blot analysis of EIF4A3 in HPCs transfected with si-EIF4A3. (I) QRT-PCR analysis of EIF4A3 and in HPCs transfected with si-circFAT1. (J) Western blot analysis of EIF4A3 in HPCs transfected with si-circFAT1. **p < 0.01, ***p < 0.001.

CircRNAs are derived from precursor RNAs through back splicing, which is associated with the typical spliceosome mechanism, involving RNA-binding proteins and complementary sequences of introns. To decipher the factors that may be involved in cyclization and biogenesis of circFAT1 cyclization, the online bioinformatics software CircInteractome was utilized to predict the RNA-binding proteins associated with circFAT1. It was found that EIF4A3 showed most putative binding sites in the back-spliced junction and the downstream flanking regions of the circFAT1 pre-mRNA (Figure 5(A)). EIF4A3 is a core component of the exon junction complex and involved in pre-mRNA splicing. EIF4A3 mRNA and protein levels were higher in HG-treated HPCs than in normal HPCs (Figure 5(B-C)). Next, we identified the potential EIF4A3 binding sites at the junction of circFAT1 as site a, and two additional putative EIF4A3 binding sites downstream of the circFAT1 pre-mRNA as sites b and c (Figure 5(D)). The RIP assay revealed that endogenous EIF4A3 could bind to the back-spliced junction site as well as the two downstream flanking regions of circFAT1 (Figure 5(E)). Furthermore, colocalization of circFAT1 and EIF4A3 in the cytoplasm was observed (Figure 5(F)), providing further support for the interaction. To determine the effect of EIF4A3 on circFAT1 production, EIF4A3 siRNAs were transfected into HPCs. As expectedlly, Knockdown of EIF4A3 resulted in decreased expression of circFAT1 in HPCs (Figure 5(G–H)). Conversely, silencing of circFAT1 had no effect on EIF4A3 expression (Figure 5(I–J)). Therefore, EIF4A3 promotes the expression of circFAT1.

### Expression of circFAT1 was elevated in DN patients

**Figure 6.**
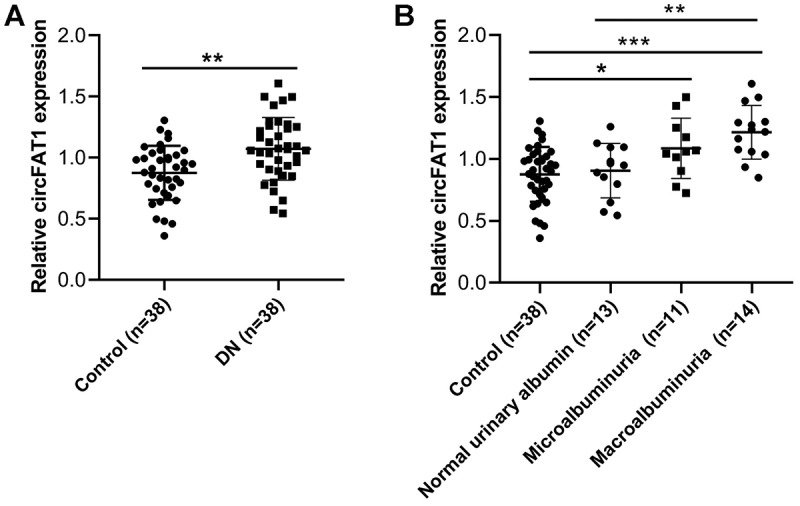
Elevated expression of circFAT1 in DN patients. (A) expression of circFAT1 in urine samples from DN patients (*n* = 38) and healthy controls (*n* = 38) were measured using qRT-PCR. (B) expression of circFAT1 in the urine samples from DN patients presenting normal urinary albumin levels, microalbuminuria, and macroalbuminuria. *p < 0.05, **p < 0.01, ***p < 0.001.

Finally, we examined the levels of circFAT1 in urine samples from DN patients. As shown in Figure 6(A), circFAT1 levels were remarkably higher in DN patients than in the control groups. We then analysed the relationship between the expression of circFAT1 and albuminuria. As presented in Figure 6(B), circFAT1 was expressed more highly in the urine samples from DN patients with microalbuminuria and macroalbuminuria than in the normal urinary albumin samples. Furthermore, our findings indicated that DN patients exhibiting increased circFAT1 expression tended to have elevated serum creatinine and cystatin-C levels, along with a decreased estimated glomerular filtration rate (Table 1).

## Discussion

The pathophysiology of DN is complex and multifaceted, involving prolonged hyperglycaemia, haemodynamic alterations, and the accumulation of advanced glycation end products, which collectively contribute to glomerular injury and fibrosis. Previous studies have revealed the role several circRNAs in DN [16–18], such as circ_0000181 and circ_0060077, which promote specific pathological processes in diabetic nephropathy [19,20]. However, the majority of circRNAs remain poorly understood. In this study, we focused on the circFAT1 and its significant role in mediating podocyte injury under HG conditions. Our results demonstrate that circFAT1 expression is markedly increased in HG-treated HPCs. Clinically, elevated circFAT1 in urine samples from DN patients correlated with albuminuria severity and renal function decline. Although our clinical cohort demonstrated a significant correlation between circFAT1 and DN markers, future large-scale studies are needed to validate its biomarker utility, particularly given the modest sample size and potential confounders like comorbidities. Silencing circFAT1 resulted in reduced podocyte apoptosis and migration, further implying its involvement in the podocyte response to hyperglycaemia. The phenomenon of EMT in podocytes is a critical event in the progression of DN. This transition is characterized by the loss of epithelial markers, such as WT-1 and Nephrin [21,22], alongside the upregulation of mesenchymal markers like FSP1 and Desmin [14]. Our findings support the association of circFAT1 with EMT, as silencing circFAT1 was correlated with decreased WT-1 and Nephrin levels and increased FSP1 and Desmin expression. This suggests that circFAT1 is indeed involved in the EMT process, contributing to podocyte dysfunction and the compromised filtration barrier associated with DN.

CircRNAs often exert their biological functions by acting as sponges for specific microRNAs (miRNAs) or RNA-binding proteins [23,24]. In this study, miR-30e-5p was identified to be likely to bind to circFAT1. Previous literature indicates that miR-30e-5p plays a protective role in podocyte injury and nephropathy model. A previous study showed that miR-30e-5p is protective in aldosterone-induced podocyte apoptosis and mitochondrial dysfunction [25]. Furthermore, Zhao D et al. [26] revealed that miR-30e was lowly expressed in the HG-stimulated renal tubular epithelial cells and renal tissues of db/db mice, and overexpression of miR-30e promoted the proliferation of RTECs and inhibit EMT. Our data showed that miR-30e-5p was downregulated in HG-stimulated HPCs. The binding of circFAT1 to miR-30e-5p was confirmed through luciferase assays and RIP experiments, solidifying its role in modulating miRNA expression.

Our study found that increased SOX4 expression, regulated positively by circFAT1 and negatively by miR-30e-5p, contributes to podocyte injury and further exacerbates the diabetic environment. The depletion of SOX4 demonstrated an increase in markers like podocin and ZO-1, highlighting its detrimental role in podocyte integrity. Furthermore, using bioinformatics tools and datasets, we predicted SOX4 as a potential target of miR-30e-5p. SOX4, known for its role in regulating embryonic development and promoting EMT in various cancers [27–29], was also shown to be upregulated in podocytes exposed to HG conditions. SOX4 depletion increased the podocin, synaptopodin, ZO-1, and P-cadherin expression and restrained the HG-induced podocyte migration [30]. Consistent with these reports, our study showed increased SOX4 expression in HPCs exposed to HG condition, negatively regulated by miR-30e-5p and positively regulated by circFAT1. In addition, SOX4 lowered the protective effect of circFAT1 deficiency on podocytes, highlighting its detrimental role in podocyte integrity.

We also explored the biogenesis of circFAT1 and identified EIF4A3, a critical RNA-binding protein, as an essential factor in promoting its production under hyperglycaemic conditions. EIF4A3, a core component of the exon junction complex, has been reported to promote the several biogenesis of circRNAs, such as hsa_circ_0001165 and circDdb1 [31,32]. Our study showed that elevated EIF4A3 levels in HG-treated HPCs facilitated the stability and formation of circFAT1, underscoring its regulatory role in circRNA biogenesis under hyperglycaemic conditions. While this study delineates the circFAT1/miR-30e-5p/SOX4 axis in podocytes, its role in vivo remains to be established. Future investigations using diabetic animal models will be essential to confirm the therapeutic potential of targeting this pathway.

Clinically, our findings regarding the detection of circFAT1 in urine samples from DN patients, particularly its correlation with the severity of albuminuria and renal function decline, highlight its potential as a non-invasive biomarker for monitoring DN progression. Unlike traditional biomarkers, the stability and specificity of circFAT1 suggest its potential utility as a non-invasive biomarker for monitoring DN progression. Further large-scale validation is needed to confirm its clinical applicability [33]. These findings align with prior studies implicating circRNAs in DN pathogenesis, expanding the understanding of their roles in regulating gene expression networks and cellular responses.

## Conclusions

In conclusion, circFAT1 plays a critical role in HG-induced podocyte injury by modulating the miR-30e-5p/SOX4 axis and is regulated by EIF4A3. Its clinical relevance as a biomarker and therapeutic target offers new avenues for the management of DN.
